# Peculiar Dental Practice

**Published:** 1869-09

**Authors:** W. H. Thrift


### PECULIAR DENTAL PRACTICE.

Dr. Livingston's report of the Batoka tribes of South-Africa, makes mention of the curious custom they have of knocking out the upper front teeth at the age of puberity. He says no young woman of this tribe thinks herself accomplished until she has got rid of the upper incisors. When questioned respecting the origin of this practice, the Batoka reply that their object is to be like oxen.

Some of the Makololo give a more facetious explanation of the custom, they say, that the wife of a chief having, in a quarrel, bitten her husband, he, in revenge, ordered her front teeth to be knocked out, and all the men in his tribe followed his example.

W. H. Thrift, D.D.S.

